# RAIM: three-stage stackelberg game for hierarchical federated learning with reputation-aware incentive mechanism

**DOI:** 10.1038/s41598-025-16830-8

**Published:** 2025-10-02

**Authors:** Cuihua Zuo, Peihua Xu, Yachen Song, Jianfeng Lu, Cao Yuan, Yaqin Li

**Affiliations:** 1 School of Mathematics and Computer Science, Wuhan Polytechnic University, Wuhan, 430024 China; 2 School of educational information technology, Faculty of Artificial Intelligence Education, Central China Normal University, Wuhan, 430079 China; 3 Hubei Meteorological Service Center, Wuhan, 430079 China; 4 School of Computer Science and Technology, Wuhan University of Science and Technology, Wuhan, 430065 China

**Keywords:** Energy science and technology, Mathematics and computing

## Abstract

Hierarchical Federated Learning (HFL) significantly enhances communication efficiency and device participation, while improving personalized learning outcomes. In this framework, incentive mechanisms are crucial as they ensure that devices actively participate and make genuine contributions. However, existing incentive mechanisms struggle to effectively address the issue of unreliable devices, which may negatively impact model training due to malicious behavior or faults, leading to low-quality updates or even failure of the global model. Additionally, participants’ strategic behaviors and device heterogeneity can further diminish the effectiveness of these mechanisms. To tackle these challenges, this paper proposes a Reputation-Aware Incentive Mechanism (RAIM) aimed at optimizing node cooperation within HFL and enhancing overall system performance. Specifically, we first evaluate the reputation value of end devices based on their training quality and historical records, which can identify and defend against malicious data attacks. Participants’ reputations are maintained through a consortium blockchain, thereby ensuring transparency and fairness. Next, we model the interaction of HFL as a three-stage Stackelberg game to address hierarchical decision-making processes, and also prove that there is a unique Stackelberg equilibrium, derived through cautiously proposed algorithms. Since the existing equilibrium may not be optimal, we further design optimal server selection algorithm to motivate high-reputation and low-cost devices to participate in training, while maximizing both system performance and social utility. Finally, extensive experiments using both synthetic and real datasets show that our RAIM outperforms state-of-the-art baseline methods.

## Introduction

With the rapid advancement of artificial intelligence (AI) and the proliferation of Internet of Things (IoT) devices, vast amounts of data are being generated at the edge of networks. Federated Learning (FL) has emerged as a promising decentralized machine learning paradigm that allows model training across distributed devices without transferring raw data to a central server^1^. This approach effectively mitigates privacy risks, reduces communication costs, and enhances system scalability, making it highly suitable for applications such as healthcare, natural language processing, autonomous driving, and security monitoring^2,3^.

Despite these advantages, FL faces several limitations in practical deployments, including high communication overhead, device dropouts, training delays, and system heterogeneity^4^. These challenges become more pronounced in large-scale scenarios where devices are resource-constrained and network conditions are unstable.

To mitigate these limitations, Hierarchical Federated Learning (HFL) has emerged as an enhanced variant of FL. HFL introduces a multi-level architecture–typically composed of cloud, edge, and end devices–where local updates are first aggregated at edge servers before being transmitted to the cloud for global optimization^5,6^. This hierarchical design improves communication efficiency by reducing the number of global communication rounds, accelerates convergence, allows edge-level personalization, and enhances system scalability. These features make HFL particularly appealing for latency-sensitive and bandwidth-constrained applications.

However, most existing HFL frameworks are built on the assumption that all clients are equally willing and able to participate in training. This assumption neglects the practical realities of device heterogeneity, energy and bandwidth constraints, and economic cost considerations, which may discourage client participation or lead to strategic behaviors^7–9^. Furthermore, traditional incentive mechanisms designed for flat FL architectures often fail to address the complex coordination dynamics introduced by hierarchical structures, such as multi-level decision-making and diverse resource availability^10,11^.

More critically, the presence of unreliable or malicious participants–who may provide poisoned, low-quality, or falsified updates either unintentionally or with adversarial intent–poses significant risks to model performance and training stability^12–14^. Ensuring robustness and fairness in such environments remains a nontrivial challenge.

To address these gaps, this study proposes a Reputation-Aware Incentive Mechanism (RAIM) tailored specifically for HFL environments. The proposed framework integrates game-theoretic modeling and reputation-based trust management to solve three major challenges: C1: Strategic Behavior. Participants may act selfishly to maximize their own benefit, such as misreporting capabilities or submitting low-quality updates, which undermines the effectiveness and fairness of the incentive mechanism^15,16^. C2: Unreliable Participants. Devices with poor behavior, whether due to adversarial attacks or unstable conditions, can negatively affect model training and must be identified and mitigated^17,18^. C3: Device Heterogeneity and Cost Disparities. The imbalance in computation and communication costs may lead some high-cost devices to lack motivation to participate^19^.

To overcome these challenges, we model the hierarchical interactions in HFL using a three-stage Stackelberg game, where the cloud, edge servers, and devices act as sequential decision-makers. A reputation evaluation module is introduced to quantify each device’s trustworthiness based on training quality and historical behavior. Reputation scores are maintained and updated via a consortium blockchain, which ensures transparency, tamper-resistance, and decentralization. By combining these components, RAIM incentivizes the participation of high-quality, low-cost devices and discourages unreliable behaviors, thus maximizing social utility and enhancing system stability.

Extensive experiments on both synthetic and real-world datasets validate the effectiveness of the proposed mechanism. Results show that RAIM significantly outperforms existing incentive schemes in terms of training efficiency, model accuracy, robustness to adversarial participants, and fairness in reward distribution.

The main contributions of this paper are as follows:

1. We propose a novel reputation-aware incentive mechanism (RAIM) to optimize node cooperation in Hierarchical Federated Learning. RAIM utilizes a reputation mechanism to identify and defend against malicious data attacks, ensuring the accuracy and convergence speed of the global model. Participants’ reputation scores are maintained through an alliance blockchain, ensuring transparency and fairness while preventing malicious behavior based on historical contribution records. RAIM designs reward mechanisms based on devices’ reputation scores and local training data volumes to incentivize high-quality device participation, thereby enhancing overall system performance. The blockchain is used to record and track participants’ reputations, ensuring the reliability and credibility of the reward mechanism.
2. We model cloud-edge-end cooperation as a three-stage Stackelberg game, decomposing it into three sub-games to address the hierarchical decision-making process. This ensures that each node maximizes its own utility while enhancing the overall social utility of the system. Additionally, we prove that this game has a unique Stackelberg equilibrium, and our designed Nash equilibrium computation algorithm effectively characterizes this equilibrium. Furthermore, each end device does not have a fixed connection to a specific edge server; instead, we design optimal server selection algorithm to motivate high-reputation, low-cost devices to participate in training, while maximizing both system performance and social utility.
3. Experiments on synthetic datasets and real-world datasets demonstrate that the proposed RAIM mechanism achieves average improvements of 16.53% and 43.21% in prediction accuracy and social utility compared to state-of-the-art methods.

The remainder of this paper is organized as follows: Related Work conducts a critical review of prior research in this domain. System Model and Problem Formulation establishes the fundamental framework and formalizes the optimization problem. Followed by the development of a reputation-aware incentive mechanism in Reputation-Aware Incentive Mechanism, we analyze the feasibility of our mechanism optand design details of imization strategies in Optimal Design of RAIM. We conduct experimental results to evaluate the mechanism’s effectiveness in Performance Evaluation, and finally conclude the paper in Conclusion.

## Related work

Federated Learning (FL) is a distributed machine learning paradigm that enables end devices to train models on local data while uploading only parameters to preserve user privacy^1^. However, traditional FL faces scalability limitations due to frequent model update communications, leading to high communication overhead, restricted participation of remote devices, degraded model generalization performance^2,3^, and challenges such as device dropout and training latency^4–6^.

To mitigate these issues, Hierarchical Federated Learning (HFL) architectures have been proposed. Literature^7^ introduced a client–edge–cloud HFL framework called HierFAVG, where multiple edge servers perform partial model aggregation to improve training efficiency and optimize the communication-computation trade-off. Literature^20^ further extended this framework by incorporating model quantization and providing a tighter convergence analysis, while optimizing aggregation intervals between client-edge and edge-cloud layers.

Literature^21^ proposed a hierarchical federated learning algorithm named FedDyn, which utilizes a dynamic weighting mechanism to suppress the divergence of local models and enhances convergence through edge-layer waiting time and group-based aggregation. Literature^22^ introduced the HierFedPDP framework that integrates a personalized differential privacy mechanism into a three-tier client–edge–cloud structure, dynamically adjusting the privacy budget based on data sensitivity to improve privacy protection and model utility. Literature^23^ focused on large-scale healthcare applications and presented an HFL framework that combines one-class SVM and LSTM to perform health trend prediction and anomaly detection from pharmacy data, enabling scalable and privacy-aware disease monitoring.

Literature^24^ proposed the FLEE framework, which deploys models on end and edge devices and enables dynamic updates using newly collected data without redeployment. Literature^25^ designed a Hybrid FL protocol that leverages dual aggregation at both the edge and cloud layers to accelerate convergence and reduce energy consumption in mobile edge computing (MEC). Literature^26^ presented PG-FFL, a reinforcement learning-based framework that automatically allocates aggregation weights to clients, improving model fairness and convergence speed.

Although these studies have advanced the performance and applicability of HFL, most of them assume universal client participation, overlooking the fact that high local training costs and device constraints may discourage engagement. This highlights the need for effective incentive mechanisms.

Recently, several works have explored incentive design in FL and HFL based on game theory, contract theory, and auction mechanisms. Literature^27^ proposed a contract-theoretic incentive mechanism that assigns rewards based on data quality and computational effort, improving aggregation accuracy. Literature^28^ combined Stackelberg games with differential privacy to balance privacy budgets and participant utility. Literature^29^ reformulated client selection as iterative subproblems solved with greedy heuristics and payment schemes. Literature^30^ modeled the HFL training process in end–edge–cloud systems as a multi-layer Stackelberg game and derived equilibrium strategies for cost-effective client contributions. However, these methods primarily focus on maximizing the utility of either the model owner or the individual participants, while neglecting global social utility and heterogeneity in client costs.

In addition, existing incentive schemes in hierarchical architectures often lack cross-layer dynamic coordination to ensure long-term engagement from reliable devices. To address this, reputation mechanisms have been introduced. Literature^31^ proposed a decentralized reputation-based personalized FL framework for MEC. Literature^32^ introduced a blockchain-assisted reputation system to improve reliability in mobile networks. Literature^33^ used reputation scores to optimize client selection under non-IID conditions, and literature^34^ combined encrypted aggregation with reputation-driven incentives for enhanced privacy and reliability. However, these works focus primarily on using reputation to filter clients rather than actively incentivizing high-reputation clients to participate.

Literature^35^ provides a tighter convergence analysis for hierarchical federated learning with model quantization and proposes optimized aggregation interval strategies to improve communication efficiency and training performance. Literature^36^ proposes a hierarchical blockchain-based federated learning architecture for connected and autonomous vehicles (CAVs), along with a supervision game–based incentive mechanism that enhances participant engagement by jointly evaluating model quality and reputation. Literature^37^ proposed a hierarchical incentive framework for federated learning that combines contract theory and Stackelberg game to address information asymmetry and incentive mismatch among task publishers, local model owners, and workers. Literature^38^ proposes a model quality maximization mechanism called MaxQ, which leverages a matching game to optimize the allocation of high-quality mobile devices to edge servers in hierarchical federated learning (HFL), aiming to improve the aggregated model performance with a proven 12-approximation ratio. Literature^39^ proposed a Quality-Aware Incentive Mechanism (QAIM) that evaluates client quality using training loss and optimizes cloud–edge–end collaboration through coalition-based Pareto improvements in a game-theoretic setting. However, it only considers short-term performance and does not account for long-term behavioral uncertainties or provide mechanisms to reward high-quality participants, thus limiting robustness and incentive sustainability.

To overcome these existing shortcomings, we propose a reputation-aware incentive mechanism based on a three-stage Stackelberg game to address the challenges posed by selfish behavior, cost differences, device heterogeneity, and unreliable participants in hierarchical federated learning model training.

## System model and problem formulation

In this section, we first introduce the hierarchical federated learning model in System Model, and then we formulate the problems in Problem Formulation.

### System model

**Fig. 1:**
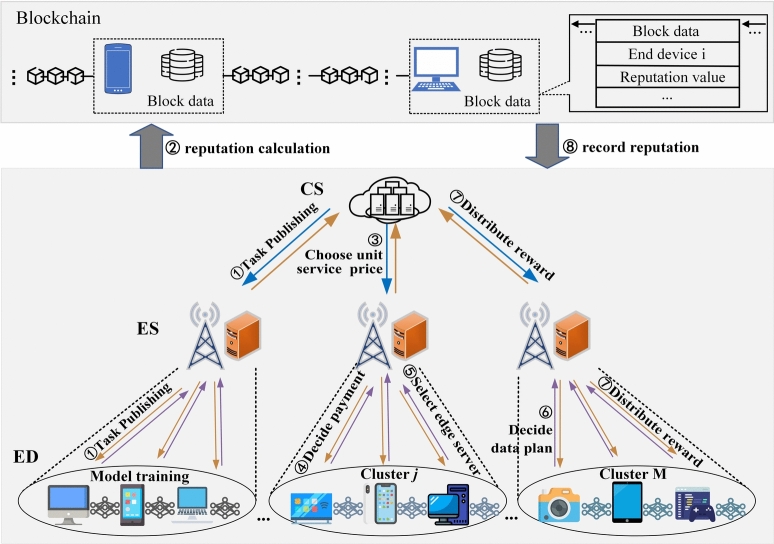
The Framework of RAIM for HFL.

As shown in Fig. 1, a HFL system model consists of the cloud server (CS), a set $\mathcal {M}=$ $\{1,2, \ldots ,$*M*$\}$ of edge servers (ESs), and *N* end devices (EDs) expressed by $\mathcal {N}=\{1$, $2, \ldots , \mathrm {\textit{N}}\}$. Each end device has its own characteristic, which is represented as a three-tuple $\left( D_i, C_i, R_i\right)$. $D_i$ is the total size of local data samples of ED *i*. $C_i$ is the unit training cost of ED *i*, and $R_i \in (0,1)$ is the reputation value of ED *i*. Each end device is free to choose which edge server to join. An ES *j* has connections with $N_j$ end devices, satisfying $N=\sum _{j=1}^M N_j$. In order to maximize the utility, ED *i* selects an optimal ES *j* and adjusts the amount of training data samples to process with the FL task by varying the ratio $\alpha _{i j}$. Conveniently, frequently used notations in the paper are listed in Table 1.

**Table 1:** Summary of notations used in this paper.

Variable	Description
*M*, *N*	Te number of edge servers, the number of end devices
*i*, $\mathcal {N}$	*i*th end device, a set of all end devices ($\mathcal {N}=\{1,2, \ldots , N\}$)
*j*, $\mathcal {M}$	*j*th edge server, a set of all edge servers ($\mathcal {M}=\{1,2, \ldots , M\}$)
$\mathcal {H}_j$	A set of EDs connected to ES *j* (also called cluster $\mathcal {H}_j$)
$\mathcal {H}_j^{+}$	S set of EDs participating in model training and connecting to ES *j*
$N_j$	The number of end devices in the cluster $H_j$ (the cardinality of $\mathcal {H}_j$)
$C_i$, $\mathcal {C}$	Unit training cost of ED *i*, $\mathcal {C}=\{C_1, C_2, \ldots , C_N\}$
$K_j$	Unit coordination and computation cost of ES *j*
$D_i$	The total size of local data of ED *i*
$R_i$, $\mathcal {R}$	The reputation value of ED *i*, $\mathcal {R}=\{R_1, R_2, \ldots , R_N\}$
$\alpha _i$, $\alpha _{ij}$, $\alpha _{-i}$	The ratio of the local training data to actual local data of ED *i*, The ratio of ED *i* when it connects to ES *j*, The strategy profile excluding $\alpha _i$
$s_i$	Edge server selection of ED *i*
*P*	The unit service pricing for the cloud server
$\gamma _j$	Reward for end devices in the cluster $\mathcal {H}_j$
$\beta$, $\lambda$	Decay factor, weighting parameter
$\delta _j$, $\theta _j$	Risk aversion parameter of ES *j*, reward scaling coefficient of ES *j*
$V_0$, $V_j$, $U_i$	Utility function of the CS, ES *j* and ED *i*

1. The cloud server: The cloud server publishes a FL task in HFL, the purpose of which is to achieve a satisfactory model performance and obtain corresponding benefit. In this article, the cloud server incentivizes edge servers through rewards and indirectly encourages end devices to participate in the model training process.
2. Edge servers: We assume that each ED can choose any ES to serve and that it does not change during the FL task once selected. As an important role in HFL, edge servers aggregate model parameters and ensure fair distribution of rewards among end devices.
3. End devices: Each ED can only select an ES to serve. If an ED *i* serves an ES *j* , we set $s_i=j$. A collection of end devices associated with the edge server *j* form the cluster $\mathcal {H}_j, \mathcal {H}_j=\left\{ i \mid i \in N \wedge s_i=j\right\}$. ED *i* selects the optimal data sample size $\alpha _i D_i\left( 0 \le \alpha _i \le 1\right)$ to participate in model training based on its unit cost ${C}_{{i}}$ and reputation ${R}_{{i}}$.
4. Blockchain: Blockchain provides a feasible solution for averting unreliable participants and preserving privacy which avoids the computational overhead and system complexity introduced by utilizing specific cryptographic algorithms. This motivates us to introduce a reputation-aware incentive mechanism with consortium blockchain that guarantees public authority and fairness. Transactions are stored in blocks containing timestamps, reputation and other information, and grow as a chain.
5. Incentive process in HFL: According to Fig. 1, the process of our system is as follows: First, the cloud server sends the global training task to the edge server and passed from it to end devices ( step 1). Next, each ES can obtain the reputation value of each ED who intends to participate in the task, based on its historical training records in consortium blockchain (step 2). After all the ESs and EDs are aware of the training task, the CS can determine the unit service price ${P}>0$ to be dispatched to ESs (step 3). Then, depending on the unit service price from the CS, each ES can decide reward declaration to attract EDs to participate in the training task (step 4). According to the published reward strategies from ESs, each ED selects an optimal ES to serve which can maximize its utility based on its unit cost and reputation value (step 5). Once all EDs have chosen their ESs based on strategy considerations, they will determine their data plan (step 6). After these steps, the HFL training process begins. After completing the training task, the CS distributes the rewards to both ESs and transmits them to EDs (step 7). Finally, the reputation value of each ED in this training task (calculated based on the credibility of the submitted parameter $\omega _{l, i}$ ) will be recorded in the blockchain (step 8).
6. Learning process in HFL: Every ED seeks to collabratively train the global model $\omega$ utilizing its local dataset. In this article, we utilize HierFAVG algorithm to update model parameters. The typical process can be summarized as below: First, the cloud server publicizes a training task, and distributes the initial global model $\omega _g^0$ to each ES. Then each ES *j* sends $\omega _g^0$ to EDs in the cluster $\mathcal {H}_j$. End devices participating in the model training train the model using their local data samples. After the first iteration of training process is completed, each participating device sends its local model $\omega _l^1$ to its ES. Each ES aggregates the received local models to generate an partial model $\omega _p^1$, which is then transmitted back to the EDs for the next training iteration. After every *k* partial model aggregations ( *k* is stipulated by the cloud server), the cloud server aggregates all the ESs’ partial models, and obtain the global model $\omega _g^1$. The process continues until the total round is reached. According to Literature^7^ the model parameters are continuously updated in a hierarchical manner: local updates $\omega _{l, i}$ from end devices are first aggregated into partial models $\omega _{p, j}$ at each edge server via Eq. 1, and then these partial models are further aggregated into a global model $\omega _g$ at the cloud server using Eq. 2.

Without loss of generality, we take a round as an example to detail the model training.

- Local training: denote ${D}_{{i}}$ as the size of local training dataset of ED *i*. During local training, its goal is to minimize the loss function $F\left( \omega _{l, i}\right)$ of model parameters $\omega _{l, i}$ based on the local dataset.
- Partial aggregation: after receiving the model updates from EDs, each ES *j* organizes partial model aggregation and obtains the aggregated new partial model $\omega _{p, j}$, which is defined as:
  1 $$\begin{aligned} \omega _{p, j}=\sum _{i \in \mathcal {H}_j} \frac{\alpha _{i j} D_i}{\sum _{i \in \mathcal {H}_j} \alpha _{i j} D_i} \omega _{l, i} \end{aligned}$$
- Global aggregation: after receiving the model updates from ESs, the cloud server conducts global model aggregation and gains the aggregated new global model $\omega _g$, which is computed as:
  2 $$\begin{aligned} \omega _g=\sum _{j=1}^M \frac{\sum _{i \in \mathcal {H}_j} \alpha _{i j} D_i}{\sum _{j=1}^M \sum _{i \in \mathcal {H}_j} \alpha _{i j} D_i} \omega _{p, j} \end{aligned}$$

The whole RAIM system enables better management of participants, ensures the quantity and quality of data contributed during model training and improves the performance of the model. In addition, it also prevents malicious participants from attacking the system.

### Problem formulation

In the process of HFL, the decision-making behaviors of nodes (CS, ESs and EDs) are sequential: The cloud server first moves, then the followers (edge servers) respond, followed by end devices taking action accordingly. Hence, we construct this interaction as a three-stage Stackelberg game model.

In the first stage, the cloud server, as a leader, publicizes a FL task and decides the corresponding unit service price *P*. In the second stage, the goal of each ES *j* is to choose a suitable reward $\gamma _j$ to attract EDs to participate in the training task and maximize its utility. To motivate EDs to engage in the model training effectively, the reward $\gamma _j$ is allocated to the EDs in proportion to the number of trustworthy training samples. In the third stage, each $\textrm{ED}$ *i*, as a follower, strategically determines the ratio $\alpha _i$ to maximize its utility. When $\alpha _i$ equals 0 , the $\textrm{ED}$ *i* will not participate in model training due to individual rationality. In other words, ED *i* will not participate in model training unless it can obtain enough payment $f_i$ to compensate for its cost $\alpha _{i j} D_i C_i$ (considering that the communication cost is much lower than the computational cost). Consequently, the utility function $U_i$ of end device *i* is the difference between the payment and cost, which can be expressed by:

3 $$\begin{aligned} U_i=U_{i j}=f_i\left( \gamma _j, \alpha _{i j}, \alpha _{-i j}\right) -\alpha _{i j} D_i C_i \end{aligned}$$

The strategy of each ES *j* is to make appropriate reward $\gamma _j$ in exchange for the participation of EDs. Traditionally, the utility of each edge *j* increases as the number of aggregated training data samples increases. However, the incremental speed should gradually diminish when the dataset gets larger, due to the generic economic law of ”diminishing marginal return”. Therefore, we define the valuation function of ES *j* to EDs’ training data in the cluster $\mathcal {H}_j$ as a sub-modular function, and materialize it as $\log _a\left( P \sum _{l \in \mathcal {H}_j^{+}} \alpha _{l j} D_l R_l+\delta _j\right)$. Then, ES *j* ’s utility function $V_j$ is formulated as:

4 $$\begin{aligned} V_j\left( r_j\right) =\log _a\left( P \sum _{l \in \mathcal {H}_j^{+}} \alpha _{l j} D_l R_l+\delta _j\right) -\frac{r_j}{\theta _j}-K_j\left| \mathcal {H}_j^{+}\right| \end{aligned}$$

where $a>1$ is a system parameter, $\mathcal {H}_j^{+}$ is the set of EDs participating in model training in the set $\mathcal {H}_j$, $\left| \mathcal {H}_j^{+}\right|$ is the number of EDs, $K_j$ is unit coordination and computation cost. The reward scaling coefficient $\theta _j$ and the risk aversion parameter $\delta _j$ are determined by each ES *j*.

The goal of the FL task is to gain a global model with the desired accuracy within a certain time period. Thus, the actual utility of the cloud server relies on the training result. Unfortunately, it is impossible to obtain the exact accuracy of a trained model before conducting the training. Since the cloud server’s utility is related to the number of training data and the reputation of EDs participating model training, the benefit of the CS can be denoted as $g\left( \sum _{j=1}^M X_j\right)$, where $X_j=\sum _{l \in \mathcal {H}_j^{+}} \alpha _{l j} D_l R_l$ is the total amount of trustworthy training data in the cluster $\mathcal {H}_j$. Due to the same reason of ”diminishing marginal return”, we define $g(x)=\lambda \ln (x+1)$, so the utility function of the CS is expressed as:

5 $$\begin{aligned} V_0(P)=\lambda \ln \left( \sum _{j=1}^M X_j+1\right) -P \sum _{j=1}^M X_j \end{aligned}$$

Finally, the social utility is defined as the sum of the utilities of all nodes, i.e.,

6 $$\begin{aligned} U^{A L L}=V_0+\sum _{j=1}^M V_j+\sum _{i=1}^N U_i \end{aligned}$$

As we known, the nodes in the HFL platform are selfish and they only care about their own utilities. Therefore, we construct a reputation-based incentive mechanism to motivate the selfish nodes to actively participate in the model training. Moreover, we model the interactive behaviors among nodes in our system as a three-stage Stackelberg game.

## Reputation-aware incentive mechanism

In this section, we design a reputation calculation rule $\mathbb {R}$, an optimal server selection rule $\mathbb {S}$, a payment rule $\mathbb {F}$, a reward rule $\mathbb {T}$ and a service pricing rule $\mathbb {P}$.

### Definition 1

(RAIM) A Reputation-Aware Incentive Mechanism is represented as a 5-tuple $(\mathbb {R}, \mathbb {S}, \mathbb {F}, \mathbb {T}, \mathbb {P})$.

- $\mathbb {R}:\left( R^{+}\right) ^t \rightarrow R^{+}$represents the reputation value of each ED.

In general, the quality of local models uploaded by FL participants affects global model performance. However, data owners may perform malicious or unreliable local model updates in order to attack platform or gain more benefits. On the one hand, malicious data owners will deliberately launch attacks, such as poisoning attacks. On the other hand, unreliable model updates are predominantly caused by false or fake dataset. The final global model accuracy and convergence speed will be affected by either malicious or unreliable model updates. Hence, we exploit the reputation mechanism to prevent malicious or unreliable data owners. Additionally, the reputation of the participant also incentives the behavior of the participant. Participants with high reputations are more likely to complete FL tasks with high quality. We use $R_i$ to represent the reputation value of the $\textrm{ED}$ *i*. If it is the first time participating in the FL platform, the reputation value is initialized to $R^0 \in (0,1)$. Otherwise, its reputation value can be calculated based on its historical reputation records in consortium blockchain.

7 $$\begin{aligned} R_i=\frac{\sum _{\tau =1}^t(1-\beta )^{t-\tau } \cdot r_i^\tau }{\sum _{\tau =1}^t(1-\beta )^{t-\tau }} \end{aligned}$$

where $\beta$ is a time decay factor and *t* is the number of times of $\textrm{ED}$ *i* participating in training within a certain period of time. $r_i^\tau \in (0,1)$ represents the reputation value of ED *i* in the $\tau$ ’th round training task. Inspired by literature^36^, it can be computed by the cosine similarity between the average local model $l_i^\tau$ and the corresponding global model $g^\tau$ as below:

8 $$\begin{aligned} r_i^\tau =\frac{\operatorname {sim}\left( l_i^\tau , g^\tau \right) +1}{2} \end{aligned}$$

9 $$\begin{aligned} \operatorname {sim}\left( l_i^\tau , g^\tau \right) =\frac{l_i^\tau \cdot g^\tau }{\left\| l_i^\tau \right\| \cdot \left\| g^\tau \right\| }=\frac{\sum _{w=1}^W\left( l_{i, w}^\tau \cdot g_w^\tau \right) }{\sqrt{\sum _{w=1}^W\left( l_{i, w}^\tau \right) ^2} \times \sqrt{\sum _{w=1}^W\left( g_w^\tau \right) ^2}} \end{aligned}$$

where $l_i^\tau$ and $g^\tau$ represent the vectors of locally average updated model parameters and globally updated model parameters, respectively. The dimensions of these two vectors are both *W*, which represents the number of total model parameters.

After completing the current training task, the reputation of ED *i* in this task is computed according to Eq. 8 and recorded in consortium blockchain.

- $\mathbb {S}: M^N \rightarrow S$ determines the optimal server selection rule, that is, which ES each ED chooses to serve, i.e.,

10 $$\begin{aligned} s_i \triangleq j \leftarrow \underset{1 \le j \le M}{\arg \max }~ U_{i j} \end{aligned}$$

- $\mathbb {F}:[0,1] \rightarrow \mathcal {R}^{+} \cup \{0\}$ represents the amount of payment each ED *i* should receive, which is proportional to the amount of local training data $\alpha _{i j} D_i$ and the reputation value $R_i$ of ED *i*, i.e.,

11 $$\begin{aligned} f_i\left( \gamma _j, \alpha _{i j}, \alpha _{-i j}\right) =\frac{\alpha _{i j} D_i R_i}{\sum _{x \in H_j} \alpha _{x j} D_x R_x} \cdot \gamma _j \end{aligned}$$

- $\mathbb {T}: \mathcal {R}^{+} \rightarrow \mathcal {R}^{+}$determines how much reward $\gamma _j$ ES *j* should pay to EDs in the cluster $H_j$, i.e.,

12 $$\begin{aligned} \gamma _j \leftarrow \underset{\gamma _j>0}{\arg \max }~ V_j \end{aligned}$$

- $\mathbb {P}: \mathcal {R}^{+} \rightarrow \mathcal {R}^{+}$decides the unit server pricing for the CS, i.e.,

13 $$\begin{aligned} P \leftarrow \underset{P>0}{\arg \max }~ V_0 \end{aligned}$$

RAIM can be regarded as a set of rules that HFL platform utilize to regulate the behaviors of its nodes. Incorporating Eqs. 3, 10 and 11, the utility maximization for each ED *i* can be written as:

14 $$\begin{aligned} \left\{ \begin{array}{l} U_i \triangleq \underset{j \in [1, M]}{\max }~ U_{i j}, \\ \text{ s.t. } \left\{ \begin{array}{l} U_{i j}=\frac{\alpha _{i j} D_i R_i}{\sum _{x \in \mathcal {H}_j} \alpha _{x j} D_x R_x} \cdot \gamma _j-\alpha _{i j} D_i C_i, \\ \alpha _{i j} \ge 0, \forall i \in [1, N] . \end{array}\right. \end{array}\right. \end{aligned}$$

Combining Eqs. 4 and 12, the utility maximization for each ES *j* can be written as:

15 $$\begin{aligned} \left\{ \begin{array}{l} V_j \triangleq \underset{\gamma _j}{\max }~ \log _a\left( P \sum _{l \in \mathcal {H}_j^{+}} \alpha _{l j} D_l R_l+\delta _j\right) -\frac{\gamma _j}{\theta _j}-K_j\left| \mathcal {H}_j^{+}\right| , \\ \text{ s.t. } \gamma _j>0, \forall j \in [1, M] . \end{array}\right. \end{aligned}$$

Combining Eqs. 5 and 13, the utility maximization for the CS can be written as:

16 $$\begin{aligned} \left\{ \begin{array}{l} V_0 \triangleq \underset{P}{\max }~ \lambda \ln \left( \sum _{j=1}^M X_j+1\right) -P \sum _{j=1}^M X_j, \\ \text{ s.t. } P>0 . \end{array}\right. \end{aligned}$$

According to RAIM, the payment received by a ED is a function of its data plan $\alpha _i$, and hence, each ED actively undertakes training tasks and take the incentive to contribute a high level effort. Furthermore, the rewards paid by each ES are directly related to the number of EDs it can attract and the amount of training data in its cluster. Meanwhile, the total training data is related to the unit server pricing. Hence, RAIM can play a positive motivating role for all nodes in HFL. Based on Eqs. 14, 15 and 16, it can be seen that RAIM always achieves a high social utility.

The RAIM design problem is defined as

17 $$\begin{aligned} \left\{ \begin{array}{l} \underset{{(\mathbb {R}, \mathbb {S}, \mathbb {F}, \mathbb {T}, \mathbb {P})} }{\max } U^{A L L} \triangleq V_0+\sum _{j=1}^M V_j+\sum _{i=1}^N U_i, \\ \text{ s.t. } \left\{ \begin{array}{l} V_0=\underset{P>0}{\max }~V_0, \\ V_j=\underset{\gamma _j>0}{\max }~ V_j, \forall j \in [1, M], \\ U_i=\underset{\alpha _j \ge 0}{\max }~ U_{i j}, \forall i \in [1, N] . \end{array}\right. \end{array}\right. \end{aligned}$$

## Optimal design of RAIM

In this section, we analyze whether there is a feasible solution to the RAIM design problem and how to find it. Then, we design an optimal server selection rule to further increase the social utility.

### Equilibrium analysis of the three-stage Stackelberg game

We begin by modeling the interactions among the cloud server, edge servers and end devices as a three-stage Stackelberg game to solve for the Nash Equilibrium (NE) solution under which the social utility can be maximized. The three-stage Stackelberg game consists of three sub-games, i.e., the Data Plan (DP) game, the Reword Declaration (RD) game and the Service Pricing (SP) game. Specifically, in the SP game, the decision of unit service pricing by the cloud server is made first, then the followers (edge servers) respond and determine appropriate rewards in the RD game, further followed by the decision of data plan by end devices in the DP game. The NE of these three sub-games may together form an Stackelberg Equilibrium (SE), which is defined as follows.

#### Definition 2

(SE) Let $P^*, {\Gamma }^*=\left( r_1^*, \cdots , r_M^*\right)$ and $\mathcal {A}^*=\left\{ \alpha _1^*, \cdots , \alpha _N^*\right\}$ be the Nash Equilibrium of the $\textrm{SP}, \textrm{RD}$ and DP game, respectively, $\left( P^*, {\Gamma }^*, \mathcal {A}^*\right)$ is an SE for the three-stage Stackelberg game if $\forall (P, {\Gamma }, \mathcal {A}) \mid \left( P \ne P^*\right) \wedge \left( {\Gamma } \ne {\Gamma }^*\right) \wedge \left( \mathcal {A} \ne \mathcal {A}^*\right)$,

18 $$\begin{aligned} \left\{ \begin{array}{l} U_i\left( P^*, {\Gamma }^*, \alpha _i^*, \alpha _{-i}^*\right) \ge U_i\left( P^*, {\Gamma }^*, \alpha _i, \alpha _{-i}^*\right) \\ V_j\left( P^*, \gamma _j^*, \gamma _{-j}^*, \mathcal {A}^*\right) \ge V_j\left( P^*, \gamma _j, \gamma _{-j}^*, \mathcal {A}^*\right) \\ V_0\left( P^*, {\Gamma }^*, \mathcal {A}^*\right) \ge V_0\left( P, {\Gamma }^*, \mathcal {A}^*\right) \end{array}\right. \end{aligned}$$

where $${j \in [1, M]}$$ and $$i \in [1, N]$$.

Finding an SE is the prerequisite for addressing Eq. 18. If the SE exists, it can be obtained by utilizing backward induction, that is, the DP game is solved first, then the RD game, and finally the SP game. In the DP game, we are trying to explore whether there is a unique Nash Equilibrium with give $\Gamma =\left\{ \gamma _1, \cdots , \gamma _M\right\}$. First, we suppose that end device *i* has already selected the edge server $s_i=j$, and its collaborators who selected the same edge server are also determined. Then end device *i* will choose its optimal training data strategy $\alpha _i^*=\alpha _{i j}^*$ to maximize its own benefit.

#### Definition 3

Given $\gamma _j, \mathcal {H}_{{j}}$, and $\alpha _{-i j}, \quad U_i\left( \alpha _{i j}^*, \alpha _{-i j}\right) \ge U_i\left( \alpha _{i j}, \alpha _{-i j}\right)$ over all $\forall \alpha _{i j} \ne \alpha _{i j}^*$.

Apparently, each strategic end device will prefer $\alpha _i^*$ in an Nash Equilibrium. In order to find the NE in the DP game, it is necessary to compute a closed-form solution of the best response strategy for each end device. The following theorem indicates that $\alpha _i^*$ is exist and unique.

#### Theorem 1

(Existence and uniqueness of NE for end devices) Given $\Gamma$ and $\mathcal {H}_j(\forall j \in [1, M])$, there is a unique NE $\mathcal {A}^*=\left( \alpha _1^*, \cdots , \alpha _N^*\right)$ in the DP game with the following properties:

1. $\left| \mathcal {H}_j^{+}\right| \ge 2$ (Note that $\mathcal {H}_j^{+}=\left\{ i \in \mathcal {H}_j \mid \alpha _{i j}>0\right\}$ represents the set of end devices which have chosen edge server *j* and participated in the training.)
2. The closed-form solution of the best response strategy can be denoted as
  19 $$\begin{aligned} \alpha _i^*=\alpha _{i j}^*= {\left\{ \begin{array}{ll}0, i \notin \mathcal {H}_j^{+} \\ \frac{\left( \left| \mathcal {H}_j^{+}\right| -1\right) \gamma _j}{D_i R_i\left( \sum _{l \in \mathcal {H}_j^{+}} \frac{C_l}{R_l}\right) ^2}\left[ \sum _{l \in \mathcal {H}_j^{+}} \frac{C_l}{R_l}-\left( \left| \mathcal {H}_j^{+}\right| -1\right) \frac{C_i}{R_i}\right] , \text{ otherwise } .\end{array}\right. } \end{aligned}$$
3. $$\forall i \in \mathcal {H}_j$$, if $$\frac{C_i}{R_i} \le \underset{l \in \mathcal {H}_j^{+}}{\max }\frac{C_l}{R_l}$$, then $$i \in \mathcal {H}_j^{+}$$.
4. $$\forall i \in \mathcal {H}_j^{+}$$, we have
  20 $$\begin{aligned} \frac{C_i}{R_i}<\frac{\sum _{l \in \mathcal {H}_j^{+}} \frac{C_l}{R_l}}{\left| \mathcal {H}_j^{+}\right| -1} \end{aligned}$$
5. All $\mathcal {H}_j^{+}(\forall j \in [1, M])$ and Nash equilibrium are unique.

#### Proof

See Proof of Theorem 1 in APPENDIX.

The above theorem reveals that the end device *i* with a normalized cost $\frac{C_i}{R_i}$ lower than $\frac{\sum _{l \in \mathcal {H}_j^+} \frac{C_l}{R_l}}{\left| \mathcal {H}_j^{+}\right| -1}$ must participate in the training. On other words, only end devices satisfying Eq. 20 will be allowed to participate in HFL model training. Thus, the incentive mechanism we designed plays an essential role in participant selection. When there are large-scale end devices in the HFL framework, the incentive mechanism selects the best-performing end devices to participate in the training, which greatly reduces cost and risk of HFL.

At Nash equilibrium, the proportion of an end device’s training data is determined by the normalized cost. We rewrite Eq. 19 as

21 $$\begin{aligned} \alpha _{i j}^*=Y_i \gamma _j, \end{aligned}$$

where

22 $$\begin{aligned} Y_i=\frac{\left( \left| \mathcal {H}_j^{+}\right| -1\right) \left[ \sum _{l \in \mathcal {H}_j^{+}} \frac{c_l}{R_l}-\left( \left| \mathcal {H}_j^{+}\right| -1\right) \frac{c_i}{R_i}\right] }{D_i R_i\left( \sum _{l \in \mathcal {H}_j^+} \frac{c_l}{R_l}\right) ^2} \end{aligned}$$

means conversion rate of payment to data (the number of trustworthy training data per unit of money). Interestingly, given a selected set $\mathcal {H}_j$, Theorem 1 indicates that there is a unique corresponding participant set $\mathcal {H}_j^{+}$, which is independent of $\gamma _j>0$ (But $\gamma _j$ can impact on the total contributed data size of the participants in $\mathcal {H}_j^{+}$).

Moreover, it is obvious that $\alpha _{i j}$ monotonically decreases with $\frac{C_i}{R_i}$. This means that the end devices with low unit cost and high reputation value are more motivated to contribute more data samples.

According to Theorem 1, we design the Nash equilibrium computing algorithms, which are elaborated in Algorithm 1. The primary idea of of Algorithm 1 is to identify the set of end devices involved based on the properties (3) and (4) of Theorem 1 (Line 6 to Line 10), and then decide each end device’s data plan $\alpha _i^*$ using property (2) (Line 11 to Line 13). The main running time of Algorithm 1 is the sorting of the fourth step, so the time complexity is $\textrm{O}(M n \log n)$, where $n=\underset{j \in [1, M]}{\max }\left| \mathcal {H}_j\right|$. $\square$

**Algorithm 1:**
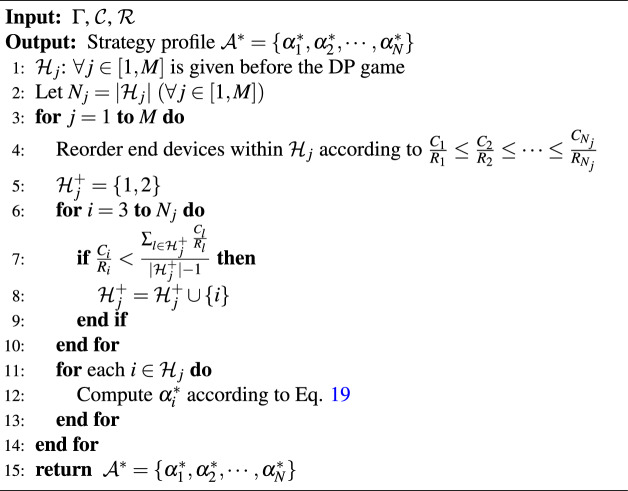
Computation of the NE for the DP Game

In the RD game, edge servers are both leaders and followers. Given the optimal response strategy $\alpha _i^*(i \in [1, N])$ of each end device, each edge server *j* needs to determine $\gamma _j$ to optimize its utility $V_j$ and balance end devices’ computation cost.

#### Definition 4

Given $\gamma _{-j}$ and a unit service price ${P},~ V_j\left( \gamma _j^*, \gamma _{-j}\right) \ge V_j\left( \gamma _j, \gamma _{-j}\right)$ over all $\forall \gamma _j \ne \gamma _j^*$.

To analyze the utility function $V_j$ for each server *j*, we substitute all $\alpha _i^*\left( i \in [1, N] \wedge s_i^*=j\right)$ into Eq. 4 to get:

23 $$\begin{aligned} V_j\left( \gamma _j\right) =\log _a\left( P B_j \gamma _j+\delta _j\right) -\frac{\gamma _j}{\theta _j}-K_j\left| \mathcal {H}_j^{+}\right| \end{aligned}$$

where $B_j=\frac{\left( \left| \mathcal {H}_j^{+}\right| -1\right) }{\sum _{l \in \mathcal {H}_j^{+}} \frac{C_l}{R_l}}$. We calculate its first-order and second-order derivatives with respect to $\gamma _j$. We obtain:

24 $$\begin{aligned} \frac{\partial V_j}{\partial \gamma _j}=\frac{P B_j}{\left( P B_j \gamma _j+\delta _j\right) \ln a}-1 / \theta _j \end{aligned}$$

and

25 $$\begin{aligned} \frac{\partial ^2 V_j}{\partial \gamma _j^2}=\frac{-P^2 B_j^2}{\left( P B_j \gamma _j+\delta _j\right) ^2 \ln a}<0 \end{aligned}$$

By setting the above first order derivative to zero, we can obtain the optimal reward strategy $\gamma _j^*$ for the edge server *j*.

26 $$\begin{aligned} \gamma _j^*=\frac{\theta _j}{\ln a}-\frac{\delta _j}{P B_j} \end{aligned}$$

Apparently, each strategic edge server will choose $\gamma _j^*$ in an Nash Equilibrium. The following theorem indicates that $\gamma _j^*$ is exist and unique.

#### Theorem 2

(Existence and uniqueness of NE for edge servers) Given a unit service price *P*, there is a unique NE $\Gamma ^*=\left\{ \gamma _1^*, \cdots , \gamma _M^*\right\}$ in the RD game.

#### Proof

See Proof of Theorem 2 in APPENDIX.

In the SP game, as the leader of the whole game, the cloud server knows that there exits NE in edge servers and end devices, so it only needs to find its optimal strategy $P^*$, which maximizes its own benefits $V_0$ and balance edge servers’ coordination cost .To analyze the utility function $V_0$ for the cloud server, we substitute all $\gamma _j^*(j \in [1, M])$ into Eq. 5 to get:

27 $$\begin{aligned} V_0(P)=\lambda \ln \left[ \sum _{j=1}^M\left( \frac{\theta _j B_j}{\ln a}-\frac{\delta _j}{P}\right) +1\right] -\sum _{j=1}^M\left( \frac{\theta _j B_j P}{\ln a}-\delta _j\right) \end{aligned}$$

We still perform the first-order derivative $\frac{\partial V_0}{\partial P}$ and make it equal to zero, then we can gain the optimal unit service price strategy $P^*$ as follows:

28 $$\begin{aligned} P^*=\frac{\sum _{j=1}^M \delta _j+\sqrt{\left( \sum _{j=1}^M \delta _j\right) ^2+4 \lambda \sum _{j=1}^M \delta _j+4 \lambda \ln a \cdot \sum _{j=1}^M \delta _j / \sum _{j=1}^M \theta _j B_j}}{2 \sum _{j=1}^M \theta _j B_j / \ln a+2} \end{aligned}$$

$$\square$$

#### Theorem 3

(Existence and uniqueness of NE for the cloud server) Given the parameters of end devices and edge servers, there exists a unique NE in the SP game.

#### Proof

See Proof of Theorem 3 in APPENDIX.

From this theorem, we can conclude that a unique NE strategy exists in the cloud server’s subgame. The cloud server does not have an intention to change its decision under the NE.

According to Definition 2, Theorem 1, Theorem 2 and Theorem 3, we know that $\left( P^*, \Gamma ^*, \Pi ^*\right)$ is a unique SE for the three-stage Stackelberg game.

### Backward induction

The constructed three-stage Stackelberg game model in our work is a large-scale nonlinear problem with a hierarchical structure. Backward induction is used to search for the dynamic game equilibrium. The so-called dynamic process shows that a sequence of actions of the players exists in the iterations, and the players who act later can observe the previous actions.

**Fig. 2:**
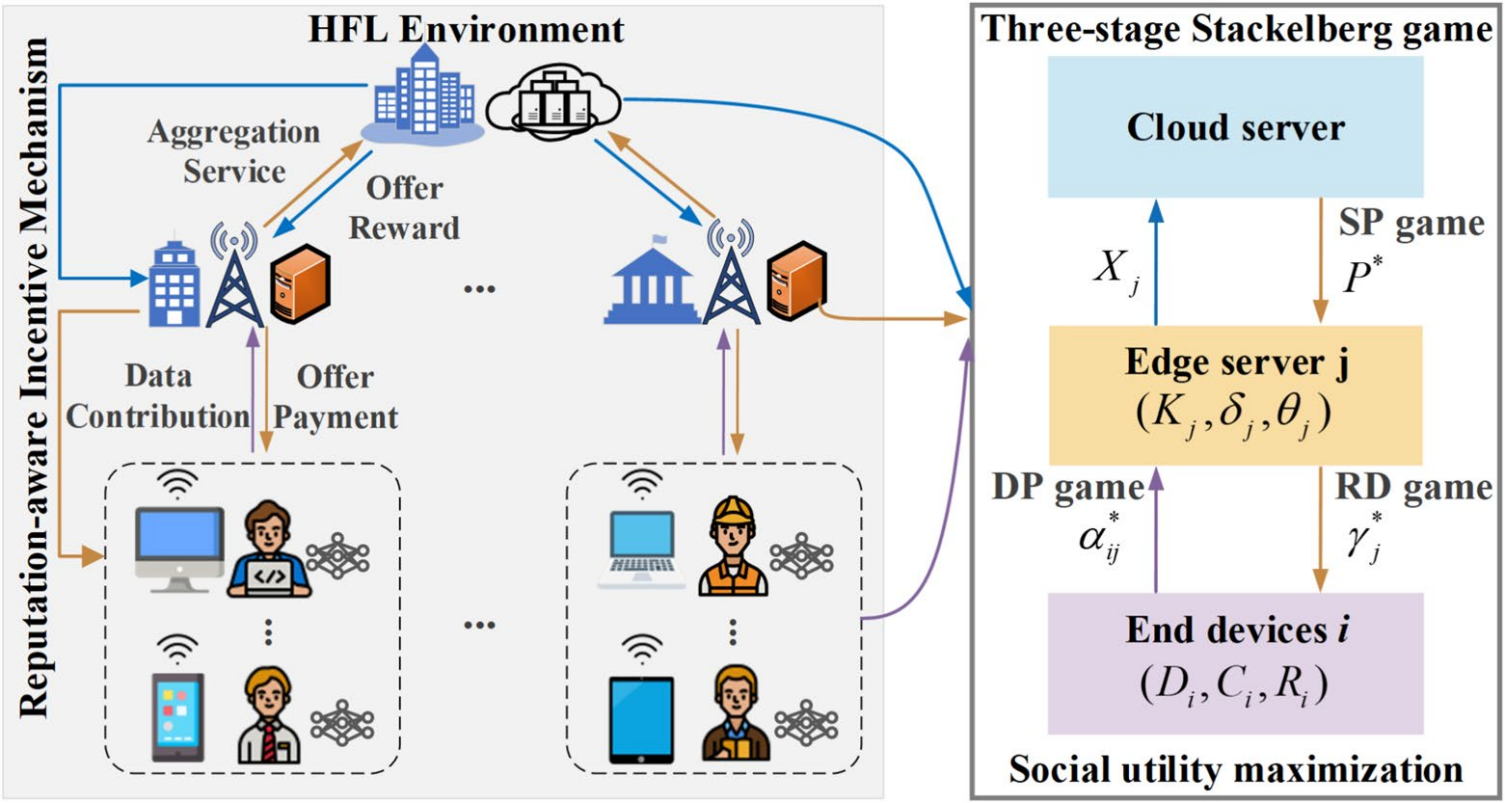
The three-stage Stackelberg game in our RAIM framework.

**Algorithm 2:**
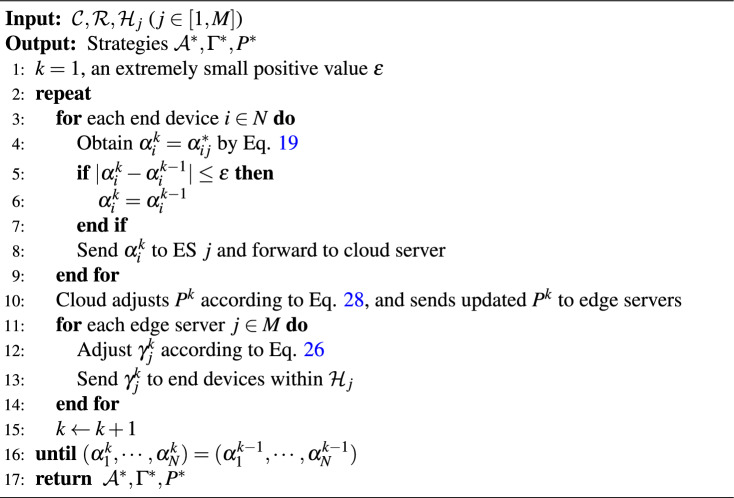
Backward Induction Algorithm to Achieve SE

In our proposed model, the leaders (the cloud server) first move and the followers (edge servers) then respond, followed by end devices taking action accordingly. If we consider the model as a combination of two two-stage Stackelberg games, it is a single leader with multi-followers and a multi-leader with multi-followers structure. The cloud server first publishes a federated learning task and initializes resource pricing strategies. The determination of the pricing strategies is affected by the behavior of edge servers and the efficiency of end devices. Therefore, we build the above policy adjustments through an iterative process. After each participant decides and takes action, others decide whether they desire to adjust their strategies to obtain a higher payment. Until all participants obtain the optimal solution, the whole system reaches equilibrium. In the three-stage Stackelberg game in Fig. 2, there is an unique NE at each stage, including the DP game of end devices, the reward strategies decided by edge servers in the RD game, and the unit service pricing determined by the cloud server in the SP game.

### Optimal design of server selection for end devices

Although given any $\mathcal {H}_j: \forall j \in [1, M]$, we can obtain a unique $\operatorname {SE}\left( \mathcal {A}^*, \Gamma ^*, P^*\right)$. However, it is may not be an optimal solution to Eq. 17. That is, the three-stage Stackelberg game only solves the equilibrium solution but does not guarantee an optimal solution for social utility. To further improve the social utility, we have designed optimal server selection algorithm for end devices, as outlined in Algorithm 3. Its key idea is to choose optimal edge server $s_i$ for each end device *i*, and its time complexity is O$(M N \log N)$.

#### Theorem 4

Given $\Gamma$, each end device’s optimal server selection strategy is fixed, relying only on their reputation and unit costs. All clusters $\mathcal {H}_j(\forall j \in [1, M])$ are the optimal and stable association.

#### *Proof*

See Proof of Theorem 4 in APPENDIX. $\square$

**Algorithm 3:**
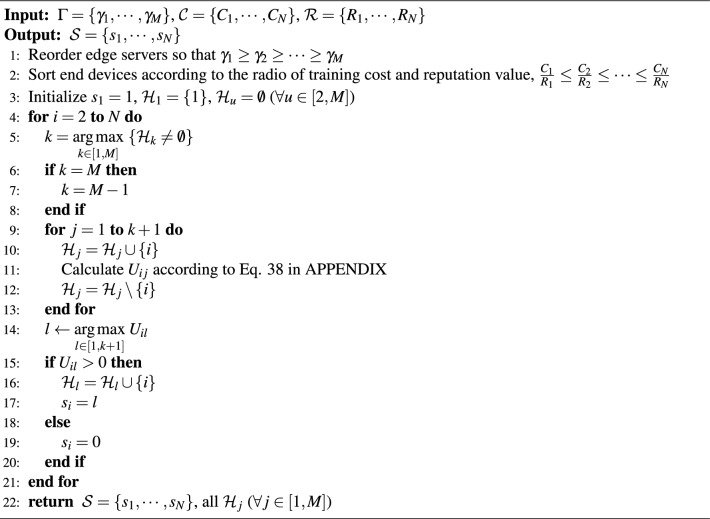
Optimal Server Selection for End Devices

## Performance evaluation

We present the performance evaluation of our designed RAIM with both synthetic and real-world datasets in this section. Specifically, we build a complete HFL system based on the FedML framework, and Pytorch is employed as the underlying training platform. The Stochastic Gradient Descent (SGD) optimizer is utilized during the training process. We use fully connected convolutional neural network (CNN) to train for all datasets.

### Experiment setup

- *Datasets:* A synthetic dataset and six real-world datasets: MNIST^40^, FEMNIST^41^, CIFAR10^42^, SVHN^43^, CINIC10^44^ and EMNIST^45^. The specific parameters of the experimental setting are shown in Table 2 and the statistics of these real-world datasets are listed in Table 3.
- *Metrics:* For the synthetic dataset, we evaluate *social utility* and *cloud server’s utility*. For the real-world datasets, we consider *prediction accuracy* and *training loss* as evaluation metrics.
- *Baselines:* The experimental parameters for all baselines are taken from the parameter settings in the corresponding literature. For a small number of variables without explicitly stated values, we use the optimal settings for all of them to ensure fairness in the comparison.

1. *FLSG*^30^: Exploits an incentive mechanism with a multi-layer game theoretical model that does not consider the reliability of end devices and the optimization if social utility.
2. *MaxQ*^38^: Presents a quality-aware incentive mechanism for HFL using a matching game.
3. *QAIM*^39^: Proposes a quality-aware incentive mechanism using Stackelberg game and coalition game.
4. *RAIM-RS*: All the methods used here are the same as those proposed in this paper, but each client only selects edge server randomly.
5. *RAIM-NO*: Utilizes the same methods as those presented in this paper, but reputation is not used in the game process. When implementing Algorithm 1 and Algorithm 3 of RAIM-NO, an average reputation value is substituted for all reputation variables.

**Table 2:** Experiment parameters.

Parameters	Values
The number of end devices *N*	[40, 100]
The number of edge servers *M*	[5, 35]
Risk aversion parameter $\delta _j$	[1, 3]
Reward scaling coefficient $\theta _j$	[1, 2]
The maximum number of global aggregations *T*	100
Edge aggregation round	5
Local update round	1
System parameter *a*	2.3
Weighting parameter $\lambda$	4
Batch size	10

**Table 3:** Statistics of the used datasets.

Dataset	Description	Classes	Image type	Train size	Test sze
MNIST	Handwritten Digits	10	Gray	60,000	10,000
EMNIST	Handwritten Digits	10	Gray	240,000	40,000
FEMNIST	Clothes & Shoes	10	Gray	60,000	10,000
CIFAR10	Vehicles & Animals	10	Color	50,000	10,000
SVHN	House Numbers	10	Color	73,257	26,032
CINIC10	Common Objects	10	Color	90,000	90,000

### Results on synthetic dataset

**Fig. 3:**
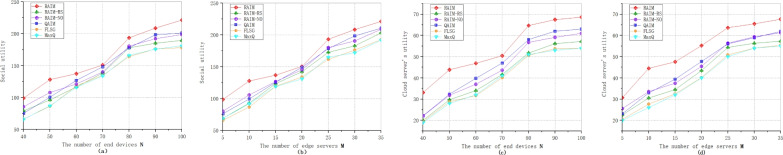
The social utility and cloud server’s utility of the compared mechanisms against *N* and *M* without unreliable end devices.

Figure 3 shows the changes in the utility of social and cloud server for the six compared mechanisms against *N* and *M*, respectively. As the number of end devices rises continuously, the social and cloud server’s utility under different mechanisms display a uniform and monotonic increase. When the number of edge servers expands, the evolution in the utility of social and cloud server exhibits a remarkably similar response. This is because the increase of nodes (including end devices and edge servers) brings more social resources to the entire system. From this figure, we sort the values of social utility in order, RAIM is the largest. On average, our proposed RAIM mechanism improves social utility by 17.87% and cloud server utility by 27.89% relative to QAIM, MaxQ and FLSG. We find the performance of FLSG and MaxQ is much lower than that of RAIM and QAIM. The fundamental reason is that FLSG does not consider clients’ training quality and MaxQ does not design client associations. Also, since end devices randomly join edge servers under the RAIM-RS mechanism, its performance is significantly lower than that of RAIM, which indicates that the optimal edge server selection algorithm plays an important role in improving performance. Without reputation in RAIM-NO, its performance is also lower than that of RAIM, which implies reputation mechanism is also crucial for improving performance. Nevertheless, the RAIM-RS and RAIM-NO mechanisms still illustrate an evident improvement compared with FLSG and MaxQ.

**Fig. 4:**
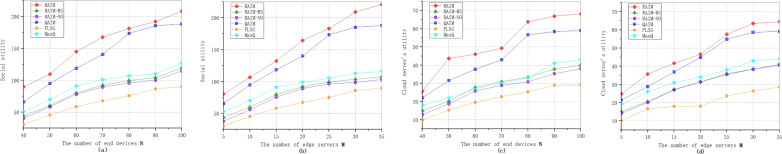
The social and cloud server’s utility of the compared mechanisms against N and M with 30% unreliable end devices.

Figure 4 demonstrates that when there are 30% unreliable end devices, as the number of end devices and edge servers increases, both the social utility and cloud server’s utility show an upward trend. In general, RAIM has the best overall performance. Compared with the results in Fig. 3, due to the influence of the incorrect labels in the local datasets of the unreliable end devices, the social and cloud server’s utility are relatively lower, especially for RAIM-NO and FLSG. This is because these two mechanisms ignore motivating reliable end devices for model training. In contrast to Fig. 3, RAIM-RS with reputation mechanism leads to a performance superiority over FLSG by a more substantial margin. MaxQ performs better than RAIM-RS and RAIM-NO because it considers the quality of model training. Oppositely, RAIM and QAIM can resist unreliable end devices well because reliable end devices are stimulated by joining optimal edge servers strategy in RAIM, and executing client associations in QAIM.

### Results on real-world datasets

**Fig. 5:**
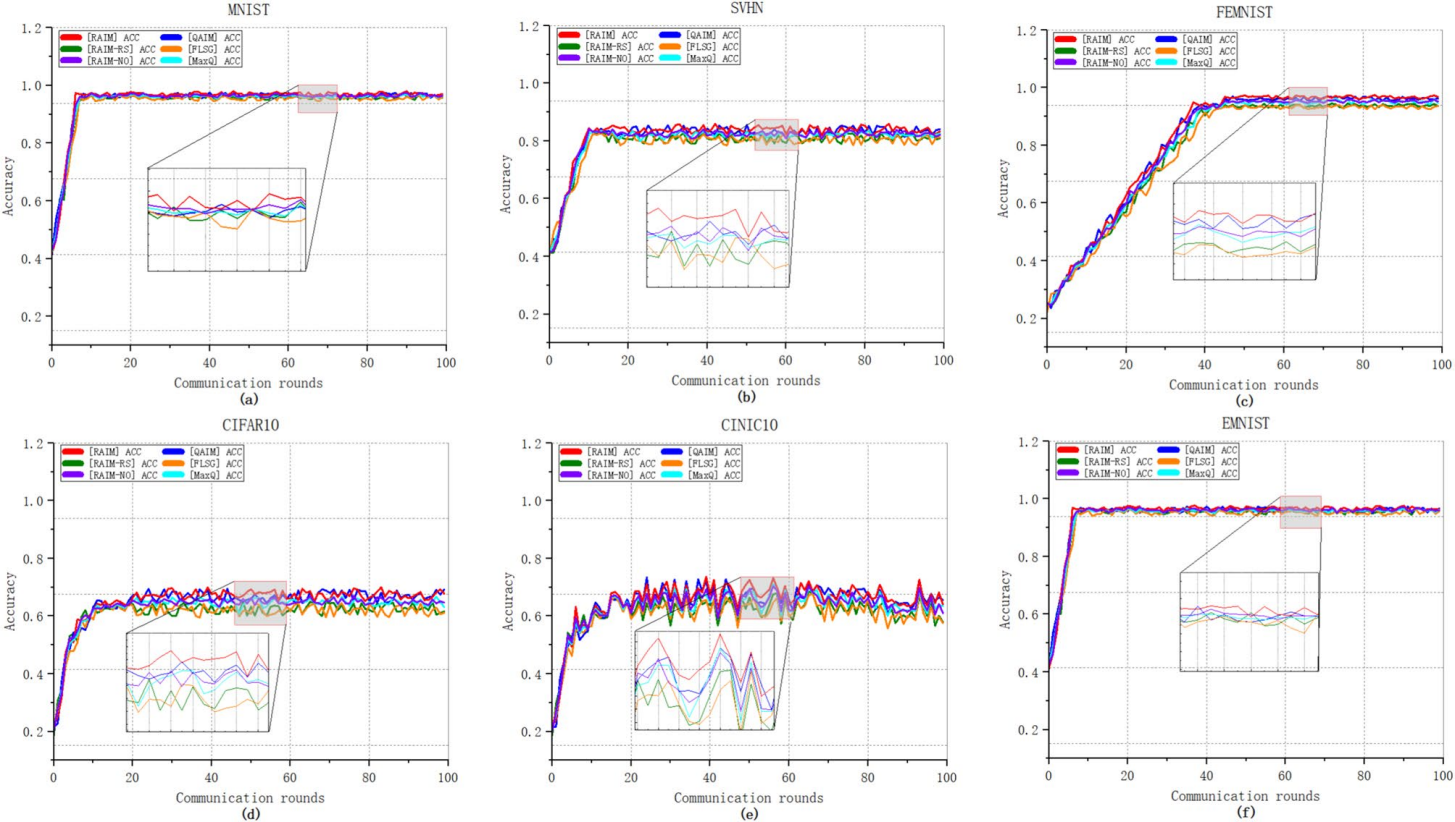
The prediction accuracy without unreliable end devices.

Figure 5 plots the prediction accuracy of six compared mechanisms in 100 rounds of global iterations on six datasets, i.e., MNIST, SVHN, FEMNIST, CINIC10, EMNIST and CIFAR10, under the condition of no unreliable end devices. It can be observed that as the number of communication rounds increases, RAIM exhibits optimal prediction accuracy on all six datasets. This indicates that RAIM demonstrates better model performance. The final global models trained by RAIM on MNIST, EMNIST and FEMNIST are closer to the 100% prediction accuracy than the models under the other five mechanisms. On datasets like SVHN, CIFAR10 and CINIC10, which are relatively more difficult to train, RAIM shows higher prediction accuracy. Moreover, we find that after the 20th round of training on CIFAR10 and the 10th round of training on SVHN, the fluctuations of RAIM-RS and FLSG are significantly greater. This is because the involvement of low-quality end devices in model training may trigger model instability. Compared to RAIM, the accuracy of RAIM-NO and RAIM-RS is relatively lower.

**Fig. 6:**
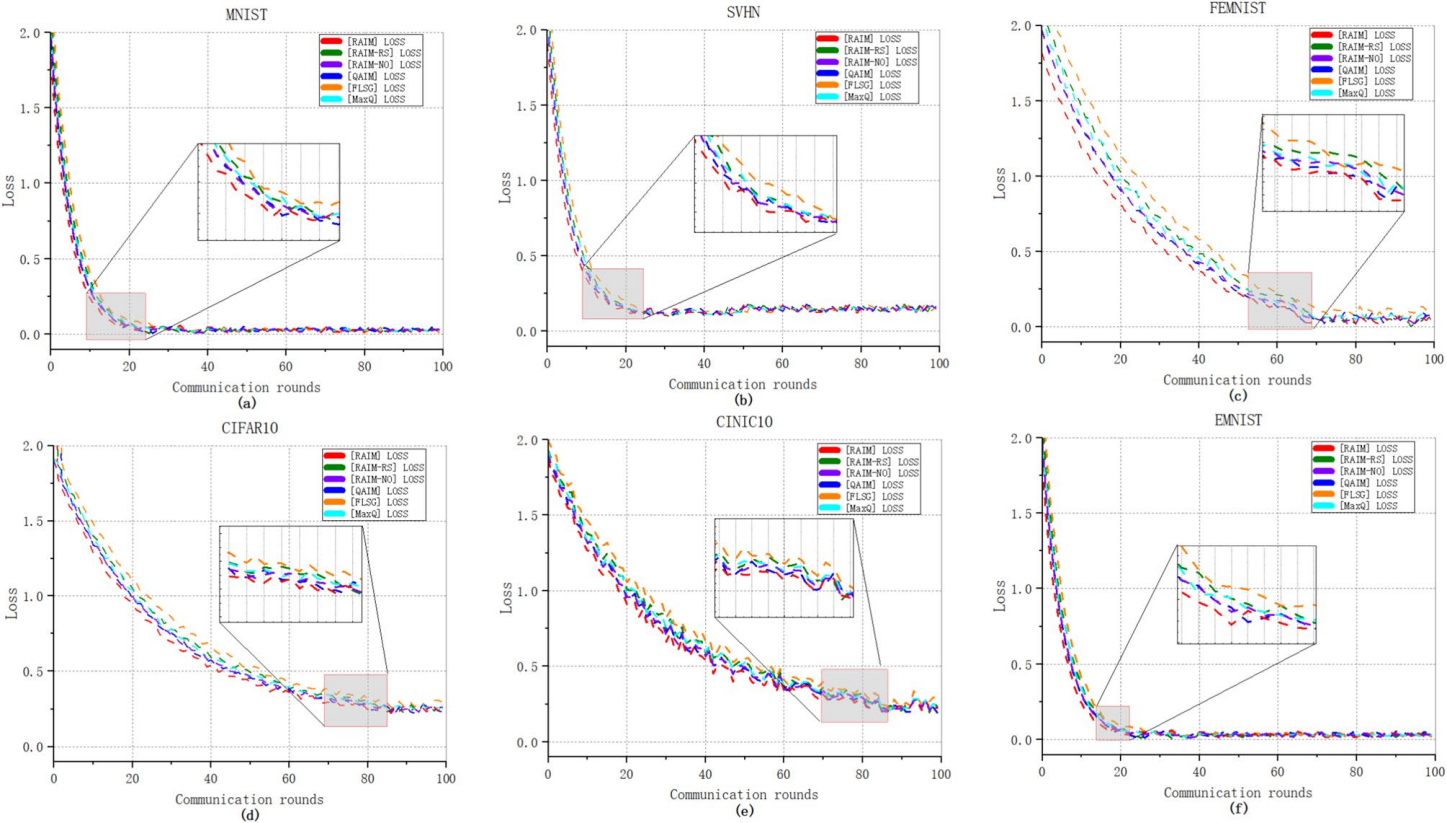
The training loss without unreliable end devices.

Figures 5 and 6 are from the same set of experiments, the major difference is that Fig. 5 focuses on prediction accuracy, while Fig. 6 focuses on training loss. Clearly, all six mechanisms achieve rapid convergence within a minimal number of communication rounds. As the number of global rounds increases, the curve of RAIM consistently descends at a faster rate, indicating that RAIM achieves a quicker convergence speed compared to the other five mechanisms. When the loss of all mechanisms stabilize during the training process, the loss values of the six mechanisms approach 0 on MNIST, EMNIST and FEMNIST datasets. Moreover, when considering the SVHN and CIFAR10 datasets, once the training process stabilizes, the overall loss value of RAIM exceeds that of the other five mechanisms slightly.

**Table 4:** Global model accuracy.

Mechanisms	RAIM			RAIM-RS			RAIM-NO		
Unreliable end devices	10%	30%	50%	10%	30%	50%	10%	30%	50%
MNIST	0.9823	0.9701	0.9350	0.9284	0.7711	0.6899	0.9201	0.7527	0.6737
EMNIST	0.9797	0.9712	0.9315	0.9258	0.7722	0.6864	0.9176	0.7496	0.6630
FEMNIST	0.9833	0.9488	0.9029	0.8311	0.7004	0.6012	0.8190	0.6990	0.5801
CIFAR10	0.6427	0.6193	0.5712	0.5723	0.5416	0.3987	0.5601	0.5412	0.3902
SVHN	0.8254	0.8031	0.7949	0.7622	0.6953	0.5928	0.7414	0.6693	0.5582
CINIC10	0.6422	0.6128	0.5710	0.5718	0.5351	0.3985	0.5675	0.5261	0.3896

**Fig. 7:**
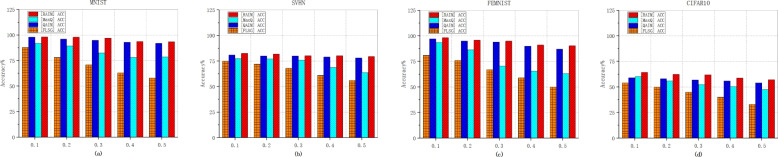
The prediction accuracy with 10%-50% unreliable end devices.

Figure 7 illustrates the prediction accuracy of the final global models for four compared mechanisms on MNIST, FEMNIST, CIFAR10 and SVHN, under the conditions where the proportions of unreliable end devices are 10%, 20%, 30%, 40%, and 50% respectively. Obviously, QAIM and RAIM, which take client incentives into account, can effectively prevent unreliable end devices from participating, thus ensuring the final quality of the global model. Furthermore, from the figure, we can find that the test accuracy of the final models of MaxQ and FLSG are more significantly affected by the incorrect data from unreliable end devices. Especially in CIFAR10, when the proportion of unreliable end devices reaches 50%, the test accuracy of the final models of the above two mechanisms reach 46.97% and 33.01% respectively, while RAIM can still achieve an accuracy of 57.12% at this time. Since QAIM also takes the quality of client and the optimization of the model performance into account, it appears quite competitive in the results of this experiment. Nevertheless, RAIM is superior to QAIM consistently across all the aforementioned datasets, which means the optimal edge server selection strategy in RAIM can better incentivize high-reputation and low-cost end devices to participate in model training.

Table 4 shows the results of the comparison of the testing accuracy of the global models trained by RAIM and two ablation studies RAIM-RS, RAIM-NO on the six real-world datasets with 10%, 30%, 50% unreliable end devices. Obviously, under any conditions, RAIM has the highest accuracy. When there are 10% unreliable end devices, the accuracy of RAIM-RS is slightly higher than that of RAIM-NO. Compared with the result of Fig. 5, RAIM-NO is more affected by unreliable end devices.

## Conclusion

In this paper, we have developed a reputation-aware incentive mechanism to stimulate participation based on a three-stage Stackelberg game. This mechanism is strategically designed to significantly enhance the model training quality of hierarchical federated learning (HFL). Specifically, we have designed and implemented three technical constituents: First, reputation storing in the consortium blockchain is used to identify and defend against malicious data attacks. Second, game theory is used to model the cloud-edge-end interaction and cooperation, and two algorithms have been proposed to calculate the unique Stackelberg equilibrium. Third, the optimal edge server selection strategy has been devised to maximize both system performance and social utility. We have conducted comprehensive experiments on both synthetic and real-world datasets. The obtained results prove the effectiveness of our RAIM mechanism in motivating participant engagement. Experiments on synthetic datasets and real-world datasets demonstrate that the proposed RAIM mechanism achieves average improvements of 16.53% in prediction accuracy and 43.21% in social utility compared to state-of-the-art methods. While our RAIM mechanism demonstrates strong performance in motivating reliable participation, it does not explicitly account for communication cost. This omission is based on the assumption that the training cost is much higher than the communication cost in many practical HFL settings. Future work will consider incorporating communication-aware factors for broader applicability. In addition, the proposed RAIM will be considered for remote sensing^46^ and medical imaging^47^, where data privacy and distributed data sources are critical. By leveraging the privacy-preserving and decentralized nature of federated learning, along with the blockchain-based reputation management and game-theoretic incentive mechanism introduced in RAIM, the system can achieve secure, trustworthy, and efficient collaborative modeling.

## Supplementary Information

Supplementary Information.

## Data Availability

The datasets analysed during the current study are available in the following repositories: MNIST: https://fedcv.s3.us-west-1.amazonaws.com/MNIST.zip. FEMNIST: https://fedml.s3-us-west-1.amazonaws.com/fed_emnist.tar.bz2. CIFAR10: https://www.cs.toronto.edu/~kriz/cifar-10-python.tar.gz. SVHN: http://ufldl.stanford.edu/housenumbers/. CINIC10: https://datashare.ed.ac.uk/handle/10283/3192. EMNIST: https://hyper.ai/cn/datasets/16446. All data and code generated during this study are freely available online at https://github.com/Sensorjang/RAIM_FedML_experiment_ZCH-master.

## References

[1] Mc Mahan, B., Moore, E., Ramage, D., Hampson, S. & y Arcas, B. A. Communication-efficient learning of deep networks from decentralized data. In *Artificial intelligence and statistics*, 1273–1282 (PMLR), (2017).

[2] Al-Rubaie, M. & Chang, J. M. Privacy-preserving machine learning: Threats and solutions. *IEEE Secur. Privacy* **17**, 49–58 (2019).

[3] Li, C., Song, M. & Luo, Y. Federated learning based on stackelberg game in unmanned-aerial-vehicle-enabled mobile edge computing. *Expert Syst. Appl.* **235**, 121023 (2024).

[4] Rieke, N. et al. The future of digital health with federated learning. *NPJ Digit. Med.* **3**, 1–7 (2020). 33015372 10.1038/s41746-020-00323-1 PMC7490367

[5] Rothchild, D. *et al.* Fetchsgd: Communication-efficient federated learning with sketching. In *International Conference on Machine Learning*, 8253–8265 (PMLR), (2020).

[6] Lim, W. Y. B. et al. Federated learning in mobile edge networks: A comprehensive survey. *IEEE Commun. Surveys Tutor.* **22**, 2031–2063 (2020).

[7] Liu, L., Zhang, J., Song, S. & Letaief, K. B. Client-edge-cloud hierarchical federated learning. In *ICC 2020-2020 IEEE International Conference on Communications (ICC)*, 1–6 (IEEE), (2020).

[8] Abad, M. S. H., Ozfatura, E., Gunduz, D. & Ercetin, O. Hierarchical federated learning across heterogeneous cellular networks. In *ICASSP 2020-2020 IEEE International Conference on Acoustics, Speech and Signal Processing (ICASSP)*, 8866–8870 (IEEE), (2020).

[9] Lim, W. Y. B. et al. Decentralized edge intelligence: A dynamic resource allocation framework for hierarchical federated learning. *IEEE Trans. Parallel Distrib. Syst.* **33**, 536–550 (2021).

[10] Donahue, K. & Kleinberg, J. Optimality and stability in federated learning: A game-theoretic approach. *Adv. Neural. Inf. Process. Syst.* **34**, 1287–1298 (2021).

[11] Wang, X. et al. Infedge: A blockchain-based incentive mechanism in hierarchical federated learning for end-edge-cloud communications. *IEEE J. Sel. Areas Commun.* **40**, 3325–3342 (2022).

[12] Le, T. H. T. et al. An incentive mechanism for federated learning in wireless cellular networks: An auction approach. *IEEE Trans. Wireless Commun.* **20**, 4874–4887 (2021).

[13] Salehi, M. & Hossain, E. Federated learning in unreliable and resource-constrained cellular wireless networks. *IEEE Trans. Commun.* **69**, 5136–5151 (2021).

[14] Posner, J., Tseng, L., Aloqaily, M. & Jararweh, Y. Federated learning in vehicular networks: Opportunities and solutions. *IEEE Netw.* **35**, 152–159 (2021).

[15] Liu, Y. et al. A secure federated learning framework for 5g networks. *IEEE Wirel. Commun.* **27**, 24–31 (2020).

[16] Ghimire, B. & Rawat, D. B. Recent advances on federated learning for cybersecurity and cybersecurity for federated learning for internet of things. *IEEE Internet Things J.* **9**, 8229–8249 (2022).

[17] Miao, Y., Liu, Z., Li, H., Choo, K.-K.R. & Deng, R. H. Privacy-preserving byzantine-robust federated learning via blockchain systems. *IEEE Trans. Inf. Forensics Secur.* **17**, 2848–2861 (2022).

[18] Deng, Y. *et al.* Fair: Quality-aware federated learning with precise user incentive and model aggregation. In *IEEE INFOCOM 2021-IEEE Conference on Computer Communications*, 1–10 (IEEE), (2021).

[19] Luo, B., Xiao, W., Wang, S., Huang, J. & Tassiulas, L. Tackling system and statistical heterogeneity for federated learning with adaptive client sampling. In *IEEE INFOCOM 2022-IEEE conference on computer communications*, 1739–1748 (IEEE), (2022).

[20] Liu, L., Zhang, J., Song, S. & Letaief, K. B. Hierarchical federated learning with quantization: Convergence analysis and system design. *IEEE Trans. Wireless Commun.* **22**, 2–18 (2022).

[21] Zhang, W., Zhao, Y., Li, F. & Zhu, H. A hierarchical federated learning algorithm based on time aggregation in edge computing environment. *Appl. Sci.* **13**, 5821 (2023).

[22] Li, S. et al. Hierfedpdp: Hierarchical federated learning with personalized differential privacy. *J. Inf. Secur. Appl.* **86**, 103890 (2024).

[23] Nariman, G. S. & Hamarashid, H. K. Hierarchical federated learning for health trend prediction and anomaly detection using pharmacy data: From zone to national scale. *Int. J. Data Sci. Anal.*10.1007/s41060-025-00756-5 (2025).

[24] Zhong, Z., Bao, W., Wang, J., Zhu, X. & Zhang, X. Flee: A hierarchical federated learning framework for distributed deep neural network over cloud, edge, and end device. *ACM Trans. Intell. Syst. Technol. (TIST)* **13**, 1–24 (2022).

[25] Wu, W., He, L., Lin, W. & Mao, R. Accelerating federated learning over reliability-agnostic clients in mobile edge computing systems. *IEEE Trans. Parallel Distrib. Syst.* **32**, 1539–1551. 10.1109/TPDS.2020.3040867 (2021).

[26] Sun, Y. *et al.* A fair federated learning framework with reinforcement learning. In *2022 International Joint Conference on Neural Networks (IJCNN)*, 1–8, 10.1109/IJCNN55064.2022.9892211 (2022).

[27] Liu, Y. *et al.* A contract theory based incentive mechanism for federated learning. In *Federated and Transfer Learning*, 117–137 (Springer, 2022).

[28] Xu, Y. et al. Incentive mechanism for differentially private federated learning in industrial internet of things. *IEEE Trans. Industr. Inf.* **18**, 6927–6939. 10.1109/TII.2021.3134257 (2022).

[29] Pang, J., Yu, J., Zhou, R. & Lui, J. C. An incentive auction for heterogeneous client selection in federated learning. *IEEE Trans. Mob. Comput.* **22**, 5733–5750 (2022).

[30] Zhao, Y. *et al.* An incentive mechanism for big data trading in end-edge-cloud hierarchical federated learning. In *2021 IEEE Global Communications Conference (GLOBECOM)*, 1–6 (IEEE), (2021).

[31] ur Rehman, M. H., Salah, K., Damiani, E. & Svetinovic, D. Towards blockchain-based reputation-aware federated learning. In *IEEE INFOCOM 2020-IEEE Conference on Computer Communications Workshops (INFOCOM WKSHPS)*, 183–188 (IEEE), (2020).

[32] Kang, J. et al. Reliable federated learning for mobile networks. *IEEE Wirel. Commun.* **27**, 72–80 (2020).

[33] Sun, K., Wu, J. & Li, J. Reputation-aware incentive mechanism of federated learning: A mean field game approach. In *2024 9th IEEE International Conference on Smart Cloud (SmartCloud)*, 48–53 (IEEE), (2024).

[34] Wang, N. et al. A blockchain based privacy-preserving federated learning scheme for internet of vehicles. *Digital Commun. Netw.* **10**(1), 126–134 (2022).

[35] Liu, L., Zhang, J., Song, S. & Letaief, K. B. Hierarchical federated learning with quantization: Convergence analysis and system design. *IEEE Trans. Wireless Commun.* **22**, 2–18. 10.1109/TWC.2022.3190512 (2023).

[36] Fu, Y., Li, C., Yu, F. R., Luan, T. H. & Zhao, P. An incentive mechanism of incorporating supervision game for federated learning in autonomous driving. *IEEE Trans. Intell. Transp. Syst.* **24**, 14800–14812 (2023).

[37] Huang, J., Ma, B., Wu, Y., Chen, Y. & Shen, X. A hierarchical incentive mechanism for federated learning. *IEEE Trans. Mob. Comput.* **23**, 12731–12747. 10.1109/TMC.2024.3423399 (2024).

[38] Hui, D., Zhuo, L. & Xin, C. Quality-aware incentive mechanism design based on matching game for hierarchical federated learning. In *IEEE INFOCOM 2022-IEEE Conference on Computer Communications Workshops (INFOCOM WKSHPS)*, 1–6 (IEEE), (2022).

[39] Hu, G. et al. Game-theoretic design of quality-aware incentive mechanisms for hierarchical federated learning. *IEEE Internet Things J.* **11**(15), 26033–26045 (2024).

[40] Lecun, Y., Bottou, L., Bengio, Y. & Haffner, P. Gradient-based learning applied to document recognition. *Proc. IEEE* **86**, 2278–2324. 10.1109/5.726791 (1998).

[41] Xiao, H., Rasul, K. & Vollgraf, R. Fashion-mnist: a novel image dataset for benchmarking machine learning algorithms. arXiv preprint arXiv:1708.07747 (2017).

[42] Krizhevsky, A., Hinton, G. *et al.* Learning multiple layers of features from tiny images (2009).

[43] Netzer, Y. *et al.* Reading digits in natural images with unsupervised feature learning. In *NIPS workshop on deep learning and unsupervised feature learning*, vol. 2011, 4 (Granada), (2011).

[44] Darlow, L. N., Crowley, E. J., Antoniou, A. & Storkey, A. J. Cinic-10 is not imagenet or cifar-10. arXiv preprint arXiv:1810.03505 (2018).

[45] Cohen, G., Afshar, S., Tapson, J. & Van Schaik, A. Emnist: Extending mnist to handwritten letters. In *2017 international joint conference on neural networks (IJCNN)*, 2921–2926 (IEEE), (2017).

[46] Ghasrodashti, E. K., Adibi, P., Karshenas, H., Kashani, H. B. & Chanussot, J. Multimodal image classification based on convolutional network and attention-based hidden Markov random field. *IEEE Trans. Geosci. Remote Sens.* **63**, 5511114 (2025).

[47] Li, X. et al. Deep learning attention mechanism in medical image analysis: Basics and beyonds. *Int. J. Netw. Dyn. Intell.* **2**, 93–116 (2023).

