# Supplementary material for: RAIM: three-stage stackelberg game for hierarchical federated learning with reputation-aware incentive mechanism
Source: Sci Rep. 2025 Oct 2;15:34344. doi: 10.1038/s41598-025-16830-8 (PMC12491596; doi:10.1038/s41598-025-16830-8)
Supplement: Supplementary file 1 — Supplementary Information. [file 41598_2025_16830_MOESM1_ESM.docx]

**APPENDIX**

1. Proof of Theorem 1

Given $\Gamma$ and $H_{j} (\forall j\in[1,M])$, we will prove the property (1) to (5) of Theorem 1.

If $\left|H_{j}^{+}\right|$= 0, obviously, each client can improve its utility from 0 to $\gamma_{j}$ by switching $\alpha_{i}$= 0 to $\alpha_{i}>0, \alpha_{i}\to0$. If $\left|H_{j}^{+}\right|$= 1, there is only one end device $k\in H_{j}^{+}$, which can also increase its utility by changing its strategy due to the lack of competitors, contradicting the NE assumption. In summary, we have $\left|H_{j}^{+}\right|\geq2$, which ends of proof of property (1).

As to the utility function $U_{ij}$of end device $i\in H_{j}$, we have $\frac{\partial U_{ij}}{\partial\alpha_{ij}}=\frac{D_{i}R_{i}\gamma_{j}}{\sum_{l\in H_{j}} \alpha_{lj}D_{l}R_{l}}-\frac{D_{i}^{2}R_{i}^{2}\alpha_{ij}\gamma_{j}}{(\sum_{l\in H_{j}} \alpha_{lj}D_{l}R_{l})^{2}}-D_{i}C_{i}$ and $\frac{\partial^{2}U_{ij}}{\partial\alpha_{ij}^{2}}=-\frac{2D_{i}^{2}R_{i}^{2}\gamma_{j}\sum_{l\in H_{j}\backslash\{i\}} \alpha_{lj}D_{l}R_{l}}{(\sum_{l\in H_{j}} \alpha_{lj}D_{l}R_{l})^{3}}$, respectively. Given any $\gamma_{j}>0$, we know that $\frac{\partial^{2}U_{ij}}{\partial\alpha_{ij}^{2}}<0$. Thus, the utility function $U_{ij}$ is strictly concave. To find the unique optimal solution, we set the first-order derivative of end devices’ utility functions as zero, i.e., $\frac{\partial U_{ij}}{\partial\alpha_{ij}}=0$. Further, we get:

$\frac{D_{i}R_{i}\gamma_{j}}{\sum_{l\in H_{j}} \alpha_{lj}D_{l}R_{l}}-\frac{D_{i}^{2}R_{i}^{2}\alpha_{ij}\gamma_{j}}{(\sum_{l\in H_{j}} \alpha_{lj}D_{l}R_{l})^{2}}-D_{i}C_{i}=0$ (29)

Because $\alpha_{ij}=0$ shows that end device $i\in H_{j}$does not participate in the training, $\sum_{l\in H_{j}^{+}} \alpha_{lj}D_{l}R_{l}=\sum_{l\in H_{j}} \alpha_{lj}D_{l}R_{l}$can be derived. Eq. 25 can be rewritten as:

$\frac{D_{i}R_{i}\gamma_{j}}{\sum_{l\in H_{j}^{+}} \alpha_{lj}D_{l}R_{l}}-\frac{D_{i}^{2}R_{i}^{2}\alpha_{ij}\gamma_{j}}{(\sum_{l\in H_{j}^{+}} \alpha_{lj}D_{l}R_{l})^{2}}-D_{i}C_{i}=0$ (30)

The solution of Eq. 30 is

$\alpha_{ij}=\left\{\begin{matrix} 0, \gamma_{j}<\frac{C_{i}}{R_{i}}\sum_{l\in H_{j}^{+}\backslash\{i\}} \alpha_{lj}D_{l}R_{l} , \\ \frac{1}{D_{i}R_{i}}(\sqrt{\frac{R_{i}\gamma_{j}\sum_{l\in H_{j}^{+}\backslash\{i\}} \alpha_{lj}D_{l}R_{l}}{C_{i}}}-\sum_{l\in H_{j}^{+}\backslash\{i\}} \alpha_{lj}D_{l}R_{l}), otherwise. \end{matrix}\right.$ (31)

By taking all end device in the set $H_{j}^{+}$ into Eq. 30 and adding them up, we further obtain an expression for the sum of all trustworthy data in the cluster H_j_:

$X_{j}=\sum_{l\in H_{j}^{+}} \alpha_{lj}D_{l}R_{l}=\frac{(\left|H_{j}^{+}\right|-1)\gamma_{j}}{\sum_{l\in H_{j}^{+}} \frac{C_{l}}{R_{l}}}$ (32)

Next, substituting Eq. 32 into Eq. 31, we get:

$\sum_{l\in H_{j}^{+}\backslash\{i\}} \alpha_{lj}D_{l}R_{l}=\frac{(\left|H_{j}^{+}\right|-1)^{2}\gamma_{j}C_{i}}{(\sum_{l\in H_{j}^{+}} \frac{C_{l}}{R_{l}})^{2}R_{i}}$ (33)

Furthermore, combining Eq. 31 and Eq. 33, we have the end device i’s strategy at Nash equilibrium:

$\alpha_{ij}^{*}=\left\{\begin{matrix} 0, i\notin H_{j}^{+} , \\ \frac{(\left|H_{j}^{+}\right|-1)\gamma_{j}}{D_{i}R_{i}(\sum_{l\in H_{j}^{+}} \frac{C_{l}}{R_{l}})^{2}}[\sum_{l\in H_{j}^{+}} \frac{C_{l}}{R_{l}}-(\left|H_{j}^{+}\right|-1)\frac{C_{i}}{R_{i}}], otherwise. \end{matrix}\right.$ (34)

Hence, the property (2) is proved. By observing the Eq. 34, when an end device i belongs to $H_{j}^{+}$, we can get:

$\frac{C_{i}}{R_{i}}<\frac{\sum_{l\in H_{j}^{+}} \frac{C_{l}}{R_{l}}}{\left|H_{j}^{+}\right|-1}$ (35)

This expression implies：

$\max_{i\in H_{j}^{+}} \frac{C_{i}}{R_{i}}<\frac{\sum_{l\in H_{j}^{+}} \frac{C_{l}}{R_{l}}}{\left|H_{j}^{+}\right|-1}$ (36)

Suppose that an end device k ($k\in H_{j} \wedge k\notin H_{j}^{+}$) satisfies $\frac{C_{k}}{R_{k}}<\max_{i\in H_{j}^{+}} \frac{C_{i}}{R_{i}}$. We can substitute it into the first derivative function as:

$\frac{\partial U_{kj}}{\partial\alpha_{kj}}=\frac{D_{k}R_{k}}{\left|H_{j}^{+}\right|-1}\sum_{l\in H_{j}^{+}} \frac{C_{l}}{R_{l}}-D_{k}C_{k} >D_{k}R_{k}(\max_{i\in H_{j}^{+}} \frac{C_{i}}{R_{i}}-\frac{C_{k}}{R_{k}})\geq0$ (37)

In this case, $\frac{\partial U_{kj}}{\partial\alpha_{kj}}>0$ means that end device k can obtain greater utility value by increasing $\alpha_{kj}$. Therefore, $\forall i\in H_{j}$, if $\frac{C_{i}}{R{}_{i}}\leq\max_{l\in H_{j}^{+}} \frac{C_{l}}{R_{l}}$, there must be $i\in H_{j}^{+}$. the properties (3) and (4) hold.

Combining the proof of properties (3) and (4), $H_{j}^{+}$ consists of a consecutive set of end devices, namely $H_{j}^{+}=\left\{W_{1},\ldots,W_{m}\right\}$, for some $m\in\left[2,N_{j}\right]$ and $\frac{C_{1}}{R_{1}}\leq...\leq\frac{C_{m}}{R{}_{m}}\leq...\leq\frac{C_{N_{j}}}{R{}_{N_{j}}}$. Notice that if $\frac{C_{x}}{R_{x}}\geq\frac{\sum_{y\in\left[1,x\right]} \frac{C_{y}}{R_{y}}}{x-1}$, then $\frac{C_{x+1}}{R_{x+1}}\geq\frac{\sum_{y\in\left[1,x+1\right]} \frac{C_{y}}{R_{y}}}{x}$. Accordingly, m must be the last index $x$ satisfying $\frac{C_{x}}{R_{x}}<\frac{\sum_{y\in\left[1,x\right]} \frac{C_{y}}{R_{y}}}{x-1}$ . Therefore, all $H_{j}^{+}(\forall j\in[1, M])$ are steady and unique. The property (5) is confirmed.

1. Proof of Theorem 2

According to Eq. 23 in the main text, we show that $V_{j}(\gamma_{j})$ is a continuous function from real number set to real number set. We can obtain that $\frac{\partial V_{j}}{\partial r_{j}}=\frac{PB_{j}}{(PB_{j}r_{j}+\delta_{j})\ln a}-\frac{1}{\theta_{j}}$ and $\frac{\partial^{2}V_{j}}{\partial r_{j}^{2}}=\frac{-P^{2}B_{j}^{2}}{(PB_{j}r_{j}+\delta_{j})^{2}\ln a}$, respectively. It is easy to judge that if P>0, then $\frac{\partial^{2}V_{j}}{\partial\gamma_{j}^{2}}<0$. Hence, the utility function $V_{j}$ is a concave function.We then know $\gamma_{j}^{*}$ exists and is unique, and the maximum value of $V_{j}$ can be computed by making $\frac{\partial V_{j}}{\partial\gamma_{j}}=0$. On the other hand, it is not difficult to determine that $\frac{\partial V_{j}}{\partial r_{j}}=\frac{PB_{j}}{(PB_{j}r_{j}+\delta_{j})\ln a}-\frac{1}{\theta_{j}}$ is differentiable on $\forall\gamma_{j}\in(0, \gamma_{j}^{*}]$, which signifies that $V_{j}$ must be continuous at this point. According to Brouwer fixed-point theorem, we know that the RD game has at least one NE. Since the utilities of all edge servers follow the incremental function until reaching a convergence, this NE is unique.

1. Proof of Theorem 3

The proof of this theorem is similar to that of Therem 2. The critical part is to prove that the utility is a concave function. According to the concave property of function $V_{0}(P)$, we can derive the optimal strategy $P^{*}$ of the cloud server by combining all$\alpha_{i}^{*} (\forall i\in[1,N])$ and $\gamma_{j}^{*} (\forall j\in[1,M])$ to solve the equation $\frac{\partial V_{0}}{\partial P}=0$. We further perform the first-order and second-order derivative as $\frac{\partial V_{0}}{\partial P}=\lambda\frac{\sum_{j=1}^{M} \delta_{j}}{\left[\sum_{j=1}^{M} (\frac{\theta_{j}B_{j}}{\ln a}-\frac{\delta_{j}}{P})+1\right]P^{2}}-\frac{\sum_{j=1}^{M} \theta_{j}B_{j}}{\ln a}$, and $\frac{\partial^{2}V_{0}}{\partial P^{2}}=\frac{-\lambda\sum_{j=1}^{M} \delta_{j}\left[(2\sum_{j=1}^{M} \theta_{j}B_{j}/\ln a+2)P-\sum_{j=1}^{M} \delta_{j}\right]}{\left[(\sum_{j=1}^{M} \theta_{j}B_{j}/\ln a+1)P^{2}-\sum_{j=1}^{M} \delta_{j}P\right]^{2}}$. From this second-order derivative expression, although it cannot be clearly seen that it is less than 0, we can prove it from another perspective. From the Eq. 5 and Eq. 27 in the main text, the function $g(x)=\lambda\ln(1+x) (\lambda>0)$ is a log function, a distinct concave function. Inside the expression, $h(P)=\sum_{j=1}^{M} (\frac{\theta_{j}B_{j}}{\ln a}-\frac{\delta_{j}}{P})$ is an obvious concave function because every parameter else is fixed and only P is a variable. Since g and h are both concave, and g is monotonically increasing function, their composite function g(h(·)) is also concave. This proves that there is a unique NE for the cloud server.

1. Proof of Theorem 4

We display that each end device $i\in N$will choose $s_{i}$ as its edge server, depending on the reputation value and unit training cost. For the convenience of description, we reorder all end devices in ascending order according to the radio of unit training cost and reputation value, $\frac{C_{1}}{R_{1}}\leq\frac{C_{2}}{R_{2}}\leq\cdot\cdot\cdot\leq\frac{C_{N}}{R_{N}}$, which will choose their edge servers in order. Given $\Gamma=\{\gamma_{1}, \cdot\cdot\cdot, \gamma_{M}\}$, let i be an end device which has just chosen edge server j with $s_{i}=j (j\in[1, M])$. By substituting Eq. 34 into Eq. 3, $U_{ij}$ can be gained when it chooses $\alpha_{ij}^{*}$.

$U_{ij}=\left\{\begin{matrix} 0, i\notin H_{j}^{+} , \\ \gamma_{j}[1-\frac{(\left|H_{j}^{+}\right|-1)\frac{C_{i}}{R_{i}}}{\sum_{l\in H_{j}^{+}} \frac{C_{l}}{R_{l}}}]^{2} , otherwise. \end{matrix}\right.$ (38)

According to the above equation, we know that $U_{ij}$ monotonically decrease with $\frac{C_{i}}{R{}_{i}}$. Meanwhile, as an end device i joins the cluster H_j_, the utility of other end devices in that cluster does not increase. Thus, for any end device k $(s_{k}\neq j)$, its optimal choice is to keep $s_{k}$ unchanged, as they clearly have no incentive to deviate from their current choice and join j instead. Without loss of generality, we assume that end device v $(s_{v}=j)$ changes its previous choice to participate in edge server u $(u\neq j)$. Now, its utility is $U_{vu}=\gamma_{u}[1-\frac{\left|H_{u}^{+}\right|\frac{C_{v}}{R_{v}}}{\sum_{l\in H_{u}^{+}} \frac{C_{l}}{R_{l}}+\frac{C_{v}}{R_{v}}}]^{2}$. If it does not deviate from edge server j, its original utility is $U_{vj}=\gamma_{j}[1-\frac{\left|H_{j}^{+}\right|\frac{C_{v}}{R_{v}}}{\sum_{l\in H_{j}^{+}} \frac{C_{l}}{R_{l}}+\frac{C_{v}}{R_{v}}}]^{2}$. If we can prove$U_{vu}\leq U_{vj}$, then the end device v will have no incentive to choose u instead of j. Because the end device i chooses j as its optimal edge server, which implies the choice j has more advantages than others at that moment, we have $\gamma_{u}[1-\frac{\left|H_{u}^{+}\right|\frac{C_{i}}{R_{i}}}{\sum_{l\in H_{u}^{+}} \frac{C_{l}}{R_{l}}+\frac{C_{i}}{R_{i}}}]^{2}\leq\gamma_{j}[1-\frac{\left|H_{j}^{+}\right|\frac{C_{i}}{R_{i}}}{\sum_{l\in H_{j}^{+}} \frac{C_{l}}{R_{l}}+\frac{C_{i}}{R_{i}}}]^{2}$. Due to $\frac{C_{v}}{R_{v}}\leq\frac{C_{i}}{R{}_{i}}$, we obtain $\gamma_{j}[1-\frac{\left|H_{j}^{+}\right|\frac{C_{i}}{R_{i}}}{\sum_{l\in H_{j}^{+}} \frac{C_{l}}{R_{l}}+\frac{C_{i}}{R_{i}}}]^{2}\leq\gamma_{j}[1-\frac{\left|H_{j}^{+}\right|\frac{C_{v}}{R_{v}}}{\sum_{l\in H_{j}^{+}} \frac{C_{l}}{R_{l}}+\frac{C_{v}}{R_{v}}}]^{2}$. In order to confirm $\gamma_{u}[1-\frac{\left|H_{u}^{+}\right|\frac{C_{v}}{R_{v}}}{\sum_{l\in H_{u}^{+}} \frac{C_{l}}{R_{l}}+\frac{C_{v}}{R_{v}}}]^{2}\leq\gamma_{j}[1-\frac{\left|H_{j}^{+}\right|\frac{C_{v}}{R_{v}}}{\sum_{l\in H_{j}^{+}} \frac{C_{l}}{R_{l}}+\frac{C_{v}}{R_{v}}}]^{2}$, we let $f(x)=\sqrt{\gamma_{j}}(1-\frac{\left|H_{j}^{+}\right|\cdot x}{\sum_{l\in H_{j}^{+}} \frac{C_{l}}{R_{l}}+x}) \text{-}\sqrt{\gamma_{u}}(1-\frac{\left|H_{u}^{+}\right|\cdot x}{\sum_{l\in H_{u}^{+}} \frac{C_{l}}{R_{l}}+x})$, which can be proven to be greater than or equal to 0 according to our previous research^1^.

Thus, we can get $U_{vu}\leq U_{vj}$, which means once an end device i choose the edge server $s_{i}$, it will have no motivation to change its choice with the joining of other end devices. Hence, all $H_{j} (\forall j\in[1, M])$ are steady and unique.

**References**

1. Lu J, Liu H, Jia R, et al. Incentivizing proportional fairness for multi-task allocation in crowdsensing[J]. IEEE Transactions on Services Computing, 2023, 17(3): 990-1000.
